# Screening and characterization of DNA aptamers that modulate prime editing

**DOI:** 10.3389/fmolb.2025.1565459

**Published:** 2025-09-09

**Authors:** Mingxia Wang, Xia Wu, Xinbo Huang, Jing Ye, Yaoting Gui

**Affiliations:** ^1^ Shenzhen Key Laboratory of Male Reproductive Medicine and Genetics, Institute of Urology, Peking University Shenzhen Hospital, Shenzhen, China; ^2^ Department of Dermatology, Institute of Dermatology, Peking University Shenzhen Hospital, Shenzhen, China; ^3^ DEYUE Skin Dermatology Clinic, Shenzhen, China

**Keywords:** CRISPR, prime editor 2, aptamer, gene editing, DNA repair

## Abstract

**Introduction:**

Precise genome editing is a critical focus in gene therapy, and the CRISPR-Cas9 system has become a powerful and versatile tool for this purpose. However, a significant limitation of the CRISPR-Cas9 system is its low homologous recombination rate, which can impede the restoration of normal gene function. To address some of these challenges, advanced gene-editing technologies, such as base editors and prime editors have been developed. Here, we explored whether Cas9-specific single-stranded DNA (ssDNA) aptamers could enhance the PE2 system’s functionality.

**Methods:**

Systematic evolution of ligands by exponential enrichment (SELEX) was utilized to isolate high-affinity Cas9-specific ssDNA aptamers. Molecular docking simulations were subsequently performed to characterize the binding interactions between these aptamers and the PE2 protein. PE2 editing efficiency was quantitatively assessed using flow cytometry and Sanger sequencing. In bladder cancer cell lines, p53 mutation repair was evaluated by quantitative PCR and Western blot analysis, while cellular responses were examined through proliferation (CCK-8) and apoptosis assays.

**Results:**

Molecular docking analysis revealed the interaction sites between SELEX-screened Cas9-specific aptamers and the PE2 protein. The incorporation of these aptamers significantly enhanced PE2 editing efficiency. In bladder cancer cells, the aptamer-PE2 complex effectively restored p53 function, leading to suppressed cellular proliferation and enhanced apoptosis rates.

**Discussion:**

Our study demonstrates that Cas9-specific aptamers can effectively enhance prime editing efficiency. This provide new insights into the modulation of prime editing and hold potential for improving its clinical applications.

## Introduction

With the growing demand for precision medicine, gene therapy has emerged as a promising approach for treating genetic disorders (Brody, 2018). Among the various genome editing platforms developed to date, CRISPR-Cas9 systems have attracted particular attention due to their versatility and relatively straightforward implementation (Pickar-Oliver and Gersbach, 2019). Numerous derivatives of this technology have already progressed to clinical testing, reflecting its transformative potential (Wang et al., 2023). However, concerns regarding off-target effects and the low efficiency of homologous recombination repair in human cells continue to constrain broader therapeutic applications of CRISPR technology (Kretzschmar et al., 2013; Ran et al., 2013). More advanced editing tools were introduced to solve these issues, such as prime editors (PE) and base editors (BE) (Kantor et al., 2020). While BEs specialize in single-nucleotide conversions, PEs offer substantially broader editing scope (Anzalone et al., 2019; Gaudelli et al., 2017).

The PE system combines a Cas9 nickase with an engineered reverse transcriptase, and is guided by a prime-editing guide RNA (pegRNA). This configuration allows for targeted nicking of the non-target DNA strand (Anzalone et al., 2019). RT elongates the nicked strand of DNA, copying sequence information from the pegRNA template to create an edited 3′flap that replaces the original genomic sequence (Ferreira da Silva et al., 2022). One of the key advantages of prime editing is that it avoids creating double-stranded breaks (DSBs), thus reducing the risks of insertions, deletions, and frameshift mutations during the DNA repair process (Daliri et al., 2024). Prime editors can induce transitions, transversions, small insertions, and deletions without the need for an exogenous donor template (Anzalone et al., 2020).

Despite these advantages, prime editing faces several challenges that limit its application, such as lower efficiency compared to other CRISPR-based technologies, delivery obstacles arise from the editor’s large size, and the prolonged time to achieve desired genetic modifications (Chen and Liu, 2023; Zhao et al., 2023). Moreover, editing outcomes can vary significantly depending on the target sequence and cell type (Chen et al., 2021). To improve the performance of prime editing, and building upon our previous studies, we hypothesized that ssDNA aptamers could modulate the activity of PE. Aptamers are known for their high specificity and offer benefits such as simple synthesis, greater stability, and better biocompatibility (Nimjee et al., 2017; Zhou and Rossi, 2017). In our previous work, we demonstrated that aptamers could modulate dCas9-mediated transcriptional regulation efficiency, as well as enhance the specificity and HDR efficiency of Cas9-mediated editing (Huang et al., 2023).

In this study, we specifically investigated whether Cas9-specific aptamers identified in our laboratory could modulate PE2 editing efficiency at both exogenous reporter loci and endogenous genomic targets. We further explored how these aptamers influence the restoration of p53 function in mutant cell lines, such as 5,637 and T24. The results showed insights into the regulation of PE2 editing and expand the toolkit for prime editing applications, with potential implications for future clinical therapies.

## Materials and methods

### Molecular docking assessment

The 3D structure of aptamer 1–5 were adopted from our previous research (Huang et al., 2023). HDOCK facilitated the prediction of docking interactions involving PE2 protein and ssDNA aptamers (Yan et al., 2017). The PE2 model used in this simulation was constructed from the structures of 4CMP (Cas9, PDB code) and 4MH8 (monomeric reverse transcriptase, PDB code), with mutations identified based on the literature (Anzalone et al., 2019). In this setup, PE2 served as the receptor, while the aptamers acted as ligands. Finally, PyMOL were utilized for 3D visualization.

### Cell lines and cell culture

Three HEK293-derived cell lines were used in this study (Brody, 2018): HEK293T cells commercially acquired from ATCC (Pickar-Oliver and Gersbach, 2019); HEK293T-GFP with AAC (510–513) deletion mutation, a generous gift from Prof. Feng Gu’s laboratory; and (Wang et al., 2023) HEK293FT-411 cells engineered by Syngentech (Beijing, China) to constitutively express TRE promoter-driven EGFP. 5,637 and T24 cells were also purchased from ATCC. Cells were maintained in DMEM supplemented with 10% FBS and antibiotics (100 U/mL penicillin +100 μg/mL streptomycin), with the exception of 5,637 cells cultured in RPMI 1640 medium.

### Plasmid construction

pegRNA plasmids were designed using the pegFinder platform developed by Sidi Chen and colleagues (Chow et al., 2020). For the exogenous GFP target, we designed pegRNAs to insert a repair template with the missing AAC sequence in the cell line (Supplementary Figure S1A). For the exogenous EGFP target, pegRNAs were created to introduce both insertion and deletion templates, allowing incorporation of a STOP codon (Supplementary Figures S1B, S1C). For endogenous targets, including EMX1 and FANCF, pegRNAs were designed with substitution templates to encompass all repair mechanisms (Supplementary Figures S1D, S1E). For the disease-related target, p53, pegRNAs were developed for the 5,637 and T24 cell lines to correct point mutations (Supplementary Figures S1F, S2A).

The PE2 plasmid used in all experiments was generously provided by David Liu through the Addgene platform (Supplementary Figure S2B). Two mutant PE2 plasmids were constructed to testify the effect of aptamer-PE2 binding sites on their function (Supplementary Figures S2C, S2D). All pegRNA plasmids were constructed by Syngentech, and the mutant PE2 plasmids were constructed by Genecefe Biotechnology (Jiangsu, China). Aptamer sequences were chemically synthesized by GenScript Biotech (Wuhan, China), featuring 5′- and 3′-end phosphorothioate modifications. Complete sequence information appears in Supplementary Table S1.

### Cell transfection

Five cell lines were plated in 12-well plates (Corning, United States) and incubated for 24 h. Transient transfection was performed using Lipofectamine 3,000 reagent (Invitrogen, United States) when cells reached 70%–80% confluence, following the manufacturer’s instructions. After 48-h transfection, the cells would undergo a 48-h puromycin selection (2 μg/mL for HEK293T cells, 1 μg/mL for T24 cells and 0.5 μg/mL for 5,637 cells). Then, the cells were collected and underwent following experiments.

### Flow cytometry assay

Fluorescence intensity quantification was performed using flow cytometry with FITC detection channels. For analysis, transfected HEK293T (GFP Del AAC) cells were digested with EDTA-free trypsin and resuspended in PBS buffer. HEK293FT-411 cells required 48-h treatment with 1 μg/mL doxycycline post-transfection before similar processing. All samples were analyzed on a Beckman CytoFlex flow cytometer (United States).

### PCR and sanger sequencing

Genomic DNA isolation from transfected cells was performed with the Ultra DNA Isolation Kit (Bei-Bei Biotech, China). PCR amplification utilized 100 ng template DNA with EmeraldAmp PCR Master Mix (TaKaRa, Japan) under the following cycling conditions: initial denaturation at 95 °C for 3 min; 30 cycles of 98 °C for 10 s, 54 °C for 30 s, and 72 °C for 25 s; final extension at 72 °C for 5 min before cooling to 4 °C. Amplified products were verified by Sanger sequencing (Sangon Biotechnology, China), and the relevant primer sequences (5′→3′) are provided below.

EMX1 Forward: TCCTGAGTTTCTCATCTGTGCC.

EMX1 Reverse: TGGTTGCCCACCCTAGTCAT.

FANCF forward: ATAGCATTGCAGAGAGGCGT.

FANCF reverse: TCTTGCCTCCACTGGTTGTG.

P53-T24 forward: CCTGAGGTGTAGACGCCAA.

P53-T24 reverse: GGCCAGACCTAAGAGCAATCA.

P53-5,637 forward: CACCTCTCATCACATCCCCG.

P53-5,637 reverse: GCCCCAATTGCAGGTAAAACA

qPCR P53-T24 forward: TGTGACTTGCACGTACTCCC

qPCR P53-T24 reverse: CTCATAGGGCACCACCACAC.

### Western blotting

Cell lysates were prepared using RIPA buffer supplemented with protease inhibitors. Protein samples (20 μg/lane) were separated by SDS-PAGE and transferred onto PVDF membranes. Following overnight incubation with primary antibodies at 4 °C, the membranes were incubated to secondary antibodies at room temperature for an hour. The protein signals were detected using an ECL chemiluminescent substrate kit (Millipore, United States). Antibody used in this study were: p53 (1:500 dilution, sc-126, Santa Cruz); β-actin (1:10000, 66009-1-PBS, Proteintech); Anti-mouse IgG (1:2000, 7076S, Cell Signalling).

### Cell apoptosis assay

Transfected cells were cultured for 48 h before H_2_O_2_ treatment, T24 cells with 0.15% H2O2 and 5,637 cells with 0.0375% H2O2 for 16 h. Then cells were harvested using EDTA-free trypsin, and then were stained with FITC-Annexin V/PI (Beyotime, China) according to the manufacturer’s protocols. The flow cytometry (Beckman CytoFlex, United States) was used to distinguish viable, early/late apoptotic, and dead cells. Relative apoptosis ratios (experimental/control) were calculated from three independent trials.

### Cell proliferation assay

Cell viability under different plasmid treatments was assessed via CCK-8 assay (Beyotime, China). Following cell counting, 3 × 10^3^ cells/well were plated in 96-well plates and allowed to adhere for 6 h. CCK-8 reagent (10 μL/well) was administered at 0, 24, 48 and 72 h time points. After 60-min incubation, absorbance at 450 nm was measured using a microplate reader (Bio-Rad, United States). Triplicate independent experiments were performed for statistical analysis.

### Statistical analysis

Triplicate biological replicates were performed for all experiments, with results expressed as mean ± standard deviations (SDs). Data were analyzed by two-way ANOVA, one-way ANOVA and unpaired t-test, and p < 0.05 was considered as statistically significant.

## Results

### Screening and verification of Cas9-specific aptamers

**FIGURE 1:**
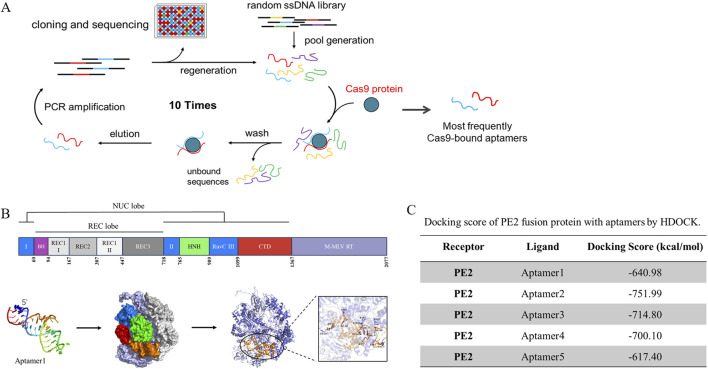
The molecular docking results of sreened aptamers with Cas9 protein. **(A)** The SELEX screening process, described in our previous study, yielded several Cas9-specific aptamers that were further utilized in this study. **(B)** Molecular docking analysis revealed the binding pose and detailed interactions of the PE2 protein with Aptamer1. The PE2 protein consists of two main components: Cas9 (dark purple) and M-MLV reverse transcriptase (light purple). Cas9 protein domains are color-coded as follows: RuvC-I, RuvC-II, and RuvC-III (blue); BH (purple); REC1 (light gray), REC2 (gray), and REC3 (dark gray); HNH (fluorescent green); and CTD (red). **(C)** The docking score between PE2 and the aptamers was calculated using HDOCK.

In the previous study, Cas9-specific ssDNA aptamers were isolated, cloned, and sequenced through the SELEX screening (Figure 1A). The top five most frequently attached aptamers continued to be used in this continuous project (Huang et al., 2023). All relevant sequences are documented in Supplementary Table S1. We employed MC-Fold/MC-Sym for aptamer 3D structure prediction, followed by PE2-aptamer docking analysis using HDOCK. Aptamer 1 was demonstrated as a representative figure (Figure 1B), The docking diagram for other aptamers were demonstrated at Supplementary Figure S3–S6. This indicated the affinity between aptamers and the PE2. Amongst them, aptamer 2, 3 and 4 displayed better docking score at −751.99, −714.8 and 700.1 kcal/mol respectively (Figure 1C).

### Stimulatory effect of Cas9-specific aptamers on PE2 on-target editing

To assess the binding of the aptamers to PE2 in a cellular context, we first evaluated their impact on the on-target editing of exogenous genes in HEK293T (GFP del AAC) and HEK293FT-411 cells. Small insertions and deletions were used to either restore GFP expression or disrupt EGFP expression. In the HEK293T (GFP del AAC) cells, pegRNA was designed to include the missing AAC sequence in the repair template. Successful editing would result in the restoration of GFP fluorescence. In HEK293FT-411 cells, pegRNAs were designed to target the few initial codons of EGFP, with the goal of inserting a STOP codon, leading to a decrease in EGFP fluorescence. The results were analyzed via flow cytometry.

For the GFP target, in the absence of aptamers, the pegRNA guided the PE2 to the GFP locus, resulting in efficient editing and detectable green fluorescence at 2.6%, which was more effective compared to the traditional Cas9 editing (Huang et al., 2023). There was no significant differences between the PE2 and the NC aptamer group. However, upon adding the Cas9-specific aptamers, we observed an increase in editing efficiency, particularly with the 75 μM aptamers 3 and 5. They managed to improve the GFP fluorescence approximately to 4.9% and 6.4% (Supplementary Figure S7).

**FIGURE 2:**
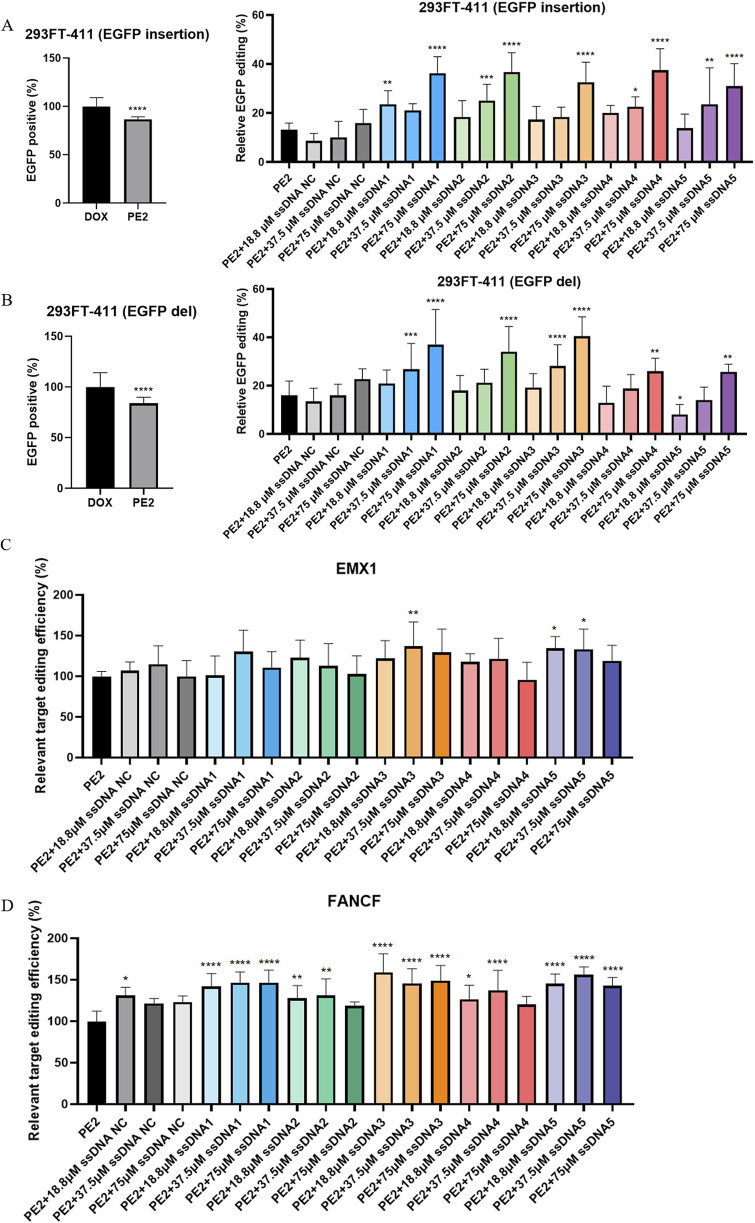
Cas9-specific aptamers enhance the on-target editing efficiency of the PE2. **(A,B)** The effect of both insertion **(A)** and deletion **(B)** PE2 repair templates on the EGFP target were analyzed by flow cytometry (left panel). Quantitative assessment of PE2 editing efficiency across different aptamer treatment groups was performed (right panel). **(C,D)** The effect of different aptamer groups on the PE2 editing efficiency on the EMX1 **(C)** and FANCF **(D)** target were assess by sanger sequencing. All editing efficiencies were normalized to the PE2-only group, which served as the negative control. ssDNA NC was another negative control where non-Cas9 specific aptamer was added. Then, aptamer 1 to aptamer 5 were presented as ssDNA1 to ssDNA5. Data were presented as mean ± SD from at least three independent experiments (*P < 0.05, **P < 0.01, ***P < 0.001).

Similarly, for the EGFP target, the PE2 system alone efficiently reduced fluorescence expression to 86.6% for insertions and 84.0% for deletions (Figures 2A,B). When Cas9-specific aptamers were introduced, a dose-dependent increase in editing efficiency was observed. While no significant differences were observed between the PE2 and the NC aptamer group, Cas9-specific aptamers significantly enhanced editing efficiency as the aptamer dose increased. For the insertion repair system, the highest relative editing efficiency reached 37.4% with 75 μM of aptamer 4 (Figure 2A; Supplementary Figure S8). In the deletion repair system, the maximum relative editing efficiency was around 40.5% with 75 μM of aptamer 3 (Figure 2B; Supplementary Figure S9).

We extended our analysis to endogenous targets, examining the effect of the PE2-aptamer system on genes EMX1 and FANCF in HEK293T cells. In this case, we focused on the single-base substitution (G- > T) repair system. While PE2 alone could effectively edit these targets, the addition of aptamers further enhanced editing efficiency. The maximum relative increase for EMX1 and FANCF editing both occurred under aptamer 3 stimulation, reaching 36.7% and 59.0% respectively (Figures 2C,D). Interestingly, no clear dose-dependency was observed in these endogenous targets, and the ability for different aptamers to facilitate the PE2 editing varies.

### Effect of Cas9-specific aptamers on PE2 mutants

The cellular experiments prompted us to explore the underlying mechanisms of aptamer-mediated modulation of PE2. Molecular docking suggested potential binding sites between the aptamers and PE2 protein (Supplementary Tables S2–S6). Among the relatively conserved interacting regions, we focused on two key residues: one in the Cas9 domain (N831) and one in the reverse transcriptase domain (K1826). We generated point mutations on the original PE2 plasmid, changing asparagine to glutamine (N831Q) and lysine to arginine (K1826R), and repeated the transfection process.

**FIGURE 3:**
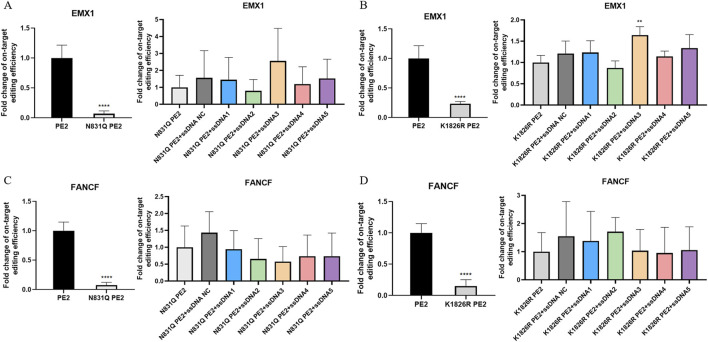
Effect of Cas9-specific aptamers on PE2 mutants. **(A,C)** Editing efficiency of the N831Q mutant PE2 was measured using Sanger sequencing on the EMX1 **(A)** and FANCF **(C)** targets. **(B,D)** Editing efficiency of the K1826R mutant PE2 was measured on EMX1 **(B)** and FANCF **(D)** targets. Editing efficiencies were standardized to the wild-type PE2 group, which served as the positive control. The effect of aptamers on mutant PE2 was also assessed on these targets, and fold change in editing efficiency was normalized to the mutant PE2 group, which served as the negative control. ssDNA NC was another negative control where non-Cas9 specific aptamer was added. Then, aptamer 1 to aptamer 5 were presented as ssDNA1 to ssDNA5. Data were presented as mean ± SD from at least three independent experiments (*P < 0.05, **P < 0.01).

Sanger sequencing revealed that the mutant PE2 proteins had significantly reduced editing function. Particularly, the N831Q mutation led to a dramatic drop in editing efficiency to less than 10% for both EMX1 and FANCF targets (Figure 3A). The K1826R mutant also showed decreased editing activity, with efficiencies of 23.3% and 15.0% for EMX1 and FANCF, respectively (Figure 3B). These results suggest that altering key amino acids in the PE2 protein significantly impairs its function. Furthermore, when aptamers were added to the system, no substantial effect on the mutant PE2’s editing activity was observed (Figure 3).

Flow cytometry analysis of EGFP protein expression further supported these findings. HEK293FT-411 cells transfected with N831Q mutant PE2 plasmids showed no significant difference in fluorescence compared to the doxycycline-treated control (Supplementary Figures S10, S11). This observation was consistent with both deletion and insertion repair template, adding aptamers to the system had no detectable effect (Supplementary Figures S10, S11). K1826R mutant PE2 shared similar findings, especially with the insertion repair template, this system had no effect on the fluorescence (Supplementary Figure S12). Yet with deletion repair template, this system significantly reduced EGFP expression to 95.8%, and aptamers 3, 4, and five were able to enhance PE2’s function in this context, with aptamer five improving relative EGFP expression to 108.8% (Supplementary Figure S13). To conclude, the overall impact of aptamers on mutant PE2 was greatly diminished, likely due to the disruption of key aptamer-PE2 binding sites.

### Cas9-specific aptamers restore the p53 expression in T24 and 5637 cell lines

**FIGURE 4:**
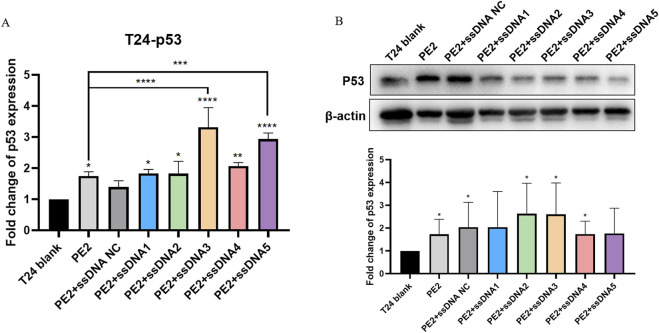
Cas9-specific aptamers influence p53 restoration in T24 cell lines. **(A)** Wild-type p53 mRNA restoration levels were measured using qPCR. **(B)** Representative Western blot image and semi-quantitative analysis of wild-type p53 protein restoration. Editing efficiencies were standardized to the PE2-only group, which acted as the negative control. ssDNA NC was another negative control where non-Cas9 specific aptamer was added. Then, aptamer 1 to aptamer 5 were presented as ssDNA1 to ssDNA5. Data were presented as mean ± SD from at least three independent experiments (*P < 0.05, **P < 0.01).

To investigate whether these findings could be translated to disease-relevant targets, we selected urinary bladder carcinoma cell lines (T24 and 5637) that harbor p53 mutations. The 5637 cell line contained the homozygous 839G>C mutation (Zhu et al., 2013), whereas the T24 cell line contained the homozygous 378C>G mutation (Makarov et al., 2017). The effect of P53 mutation in T24 (378C>G) causes an in-frame deletion of tyrosine 126, and the effect of P53 mutation in 5673 (839G>C) is a missense mutation (R280T). In both cases, the cell lines loses normal P53 function. We designed pegRNAs that were specific to the mutated p53 locus to restore wild-type p53 expression. In T24 cells co-transfected with PE2/pegRNA (p53) and aptamers, qPCR results suggested that PE2 could significantly enhance the restoration of p53 mRNA expression level by 1.75-fold. In addition, aptamers could facilitate this restoration. Specifically with aptamer 3 and 5, the p53 mRNA expression was increased to 3.32 and 2.94-fold, respectively (Figure 4A). Western blot analysis showed that PE2 significantly elevated p53 protein expression by 1.73-fold. While aptamers further facilitated this restoration, no significant difference was observed between the aptamer-treated and PE2-only groups (Figure 4B).

**FIGURE 5:**
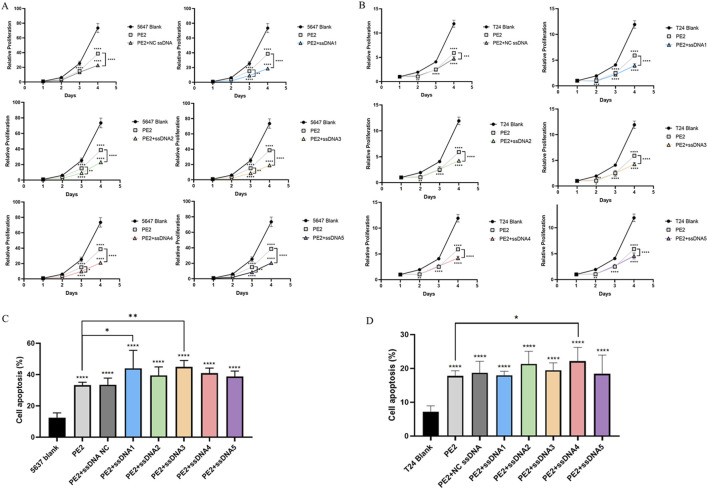
Restoring p53 affects the cell function of 5,637 and T24 cells. **(A,B)** CCK-8 assay showing the effect of aptamers on PE2-mediated cell proliferation in 5,637 and T24 wild-type p53-deficient cells. **(C,D)** The effect of aptamers on PE2-mediated cell apoptosis was assessed by flow cytometry in 5,637 and T24 wild-type p53-deficient cells. ssDNA NC was another negative control where non-Cas9 specific aptamer was added. Then, aptamer 1 to aptamer 5 were presented as ssDNA1 to ssDNA5. Data were presented as mean ± SD from at least three biological replicates (*P < 0.05, **P < 0.01, ***P < 0.001).

Next, we evaluated whether restoring wild-type p53 expression could inhibit the proliferation of p53-deficient cancer cells. CCK-8 assays demonstrated that PE2-treated groups exhibited significantly reduced cell proliferation compared to controls. Aptamer-treated groups showed even greater inhibition of cell growth than the PE2-only group, highlighting the role of p53 restoration in suppressing tumor cell growth (Figures 5A,B). A similar enhancement was observed in the NC aptamer group, this could be explained by Wang et al., Lipofectamine 3000 reagent exhibits significant cytotoxicity. The addition of non-specific ssDNAs further exacerbated cellular damage, decreasing cell viability upon transfection (Wang et al., 2018). Though NC aptamer exerted an effect on cell growth, target-specific aptamers exhibited more pronounced effects (Figures 5A,B).

Furthermore, PE2 treatment markedly promoted apoptosis in both 5,637 (2.65-fold) and T24 cells (2.30-fold) compared to the blank control (Figures 5C,D; Supplementary Figures S14, S15). Aptamer-treated groups further promoted apoptosis, with aptamers 1 and aptamers 3 showing significant effects in 5,637 cells. They enhanced the apoptosis of 5,637 cells to 3.51-fold and 3.59-fold respectively compared to the blank control, and 1.32-fold and 1.35-fold compared to the PE2 only group (Figure 5C). Aptamer 4 performed best in T24 cells, which increased the apoptosis to 3.09-fold compared to the blank control and 1.25-fold compared to the PE2 only group (Figure 5D). These results suggest that the PE2-aptamer system can restore wild-type p53 expression and induce apoptosis in p53-deficient cancer cells.

## Discussion

The low editing efficiency of PEs remains a significant obstacle, limiting their broader application (Yu et al., 2023). In efforts to enhance prime editing, researchers have approached the problem from two key perspectives: modifying the pegRNA and optimizing the protein components of prime editors (Chen et al., 2021; Nelson et al., 2021; Zong et al., 2022). Improvements on pegRNA have included methods such as increasing expression through polycistronic tRNAs and ribozymes (Jiang et al., 2020; Lin et al., 2020), designing pegRNAs with optimal melting temperatures (Lin et al., 2021), utilizing dual-pegRNA systems (Lin et al., 2021), and stabilizing pegRNAs with engineered variants (Nelson et al., 2021). On the protein side, the development of PE2 marked a significant leap, as it introduced mutations that increased editing efficiency (Anzalone et al., 2019). Subsequent versions like PE3, PE4, and PE5 involved the addition of sgRNAs or negative mismatch repair proteins, such as MLH1, to further enhance editing efficiency (Chen et al., 2021; Doman et al., 2022; Yan et al., 2024). Other improvement involved protein engineering also aimed to enhance efficiency (Zong et al., 2022). These updates have shown variable success across different cell types and targets (Anzalone et al., 2019; Anzalone et al., 2020; Lin et al., 2020), yet they primarily focus on altering the core editing components.

We opted not to explore PE3, PE4, or PE5 in our study for several reasons. While these variants have shown improvements in the editing efficiency, they introduce additional layers of complexity. For example, PE3 would create a secondary nick on the unedited strand, which increased the risk of creating a staggered DSB (Truong et al., 2024). For PE4 and PE5, the additional MLH1 increases the number of variables in the editing process, together with different concentrations of aptamer may induce extra cellular stress during transfection (Fiszer-Kierzkowska et al., 2011). In addition, the performance of the updated PEs varies across different cell lines and gene targets (Chen et al., 2021), their application in routine gene editing is more complicated. Instead, we aimed to explore a simpler and potentially more universal method for improving prime editing, focusing on Cas9-specific aptamers as a regulatory element that could be easily introduced into the system.

In summary, we demonstrated that Cas9-specific ssDNA aptamers can enhance PE2-mediated gene editing. This enhancing effect varies based on different targets or aptamers added. This observation was supported in previous publications (Anzalone et al., 2019; Petri et al., 2022), and could be due to several reasons, including most commonly, the difference in sgRNA design (Moreno-Mateos et al., 2015). The specificity of spacer region, the GC content, the secondary structure of sgRNA could all affect their binding towards target DNA (Dang et al., 2015). In addition, chromatin accessibility is also a key factor influencing editing efficiency. As Verkuijl SA, et al. suggested, the better the chromatin accessibility, the higher the CRISPR editing efficiency (Verkuijl and Rots, 2019). Thus, experimental validation is required to determine the optimal editing site and the most effective aptamer at the target locus.

Subsequently, we investigated the potential mechanisms underlying the impact of aptamers on PE2 function. The findings revealed that mutation of the relatively conserved binding sites abolished the enhancing effect of aptamers on PE2 activity. As previous literature suggested, aptamer-protein binding could lead to a minor protein conformational change (Shraim et al., 2022). Under appropriate 3D structural conditions, aptamer-PE2 binding may facilitate gene editing process. More importantly, we applied this system to cancer cell lines, showing that aptamers can help correct mutant genes. This has potential implications for future cancer treatments and the correction of other genetic diseases. Our results introduce an innovative strategy for modulating prime editing and extending the regulatory toolbox available for CRISPR-based systems.

## Data Availability

The original contributions presented in the study are included in the article/Supplementary Material, further inquiries can be directed to the corresponding author.
