# Supplementary material for: Screening and characterization of DNA aptamers that modulate prime editing
Source: Front Mol Biosci. 2025 Sep 9;12:1565459. doi: 10.3389/fmolb.2025.1565459 (PMC12454028; doi:10.3389/fmolb.2025.1565459)
Supplement: Supplementary file 1 [file Supplementaryfile1.docx]

**
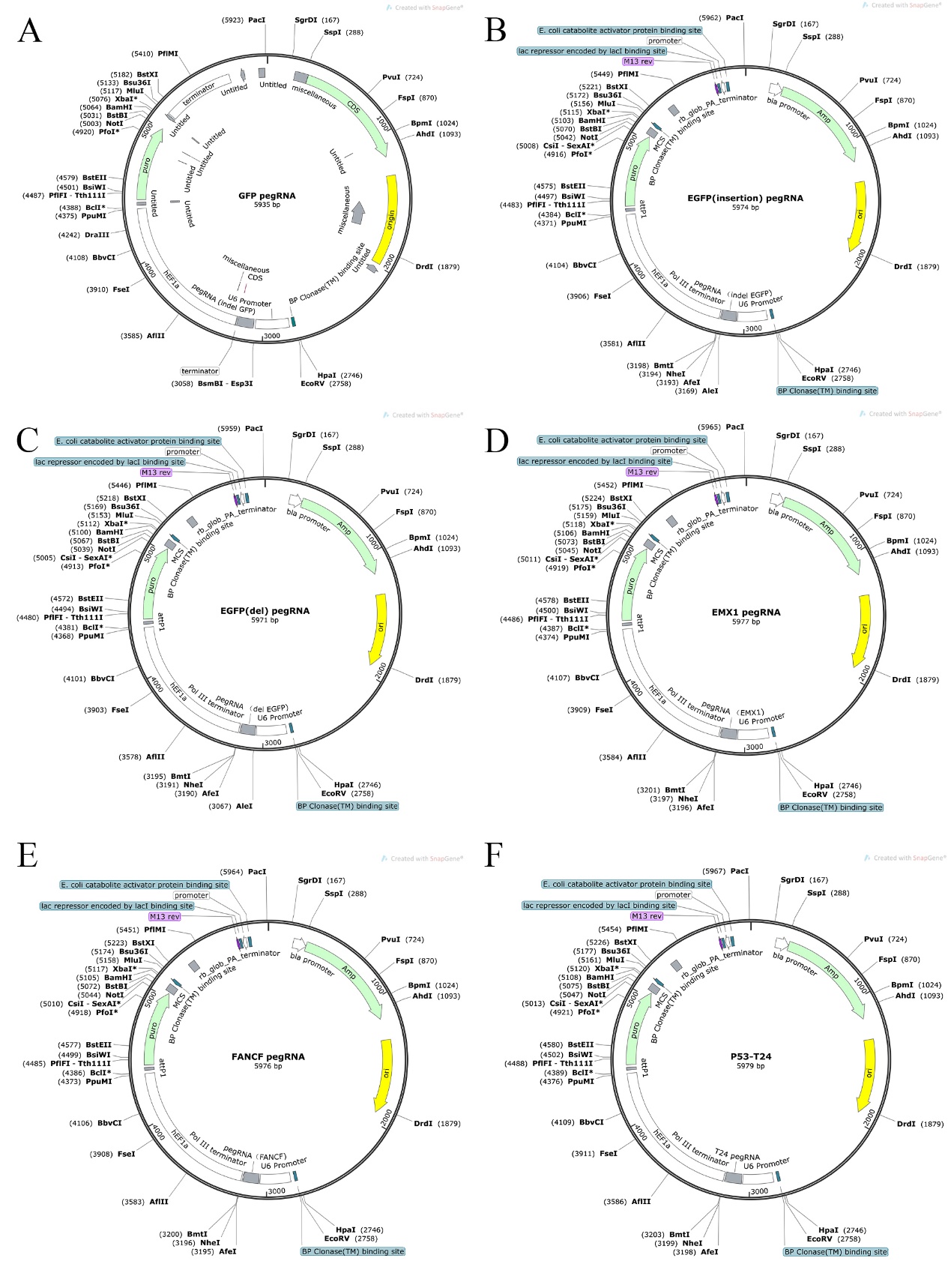
**

**Figure S1: Related plasmids.** (A) pZDonor_Seq1-U6-pegRNA(GFP)-hEF1a-Puro-Seq2. (B) pZDonor_Seq1-U6-pegRNA (insertion EGFP)-terminator-hEF1a-Puro-Seq2. (C) pZDonor_Seq1-U6-pegRNA(deletion EGFP)-terminator-hEF1a-Puro-Seq2. (D) pZDonor_Seq1-U6-pegRNA (EMX1)-terminator-hEF1a-Puro-Seq2. (E) pZDonor_Seq1-U6-pegRNA(FANCF)-terminator-hEF1a-Puro-Seq2. (F) pZDonor_Seq1-U6-T24 pegRNA(p53)-terminator-hEF1a-Puro-Seq2.

**
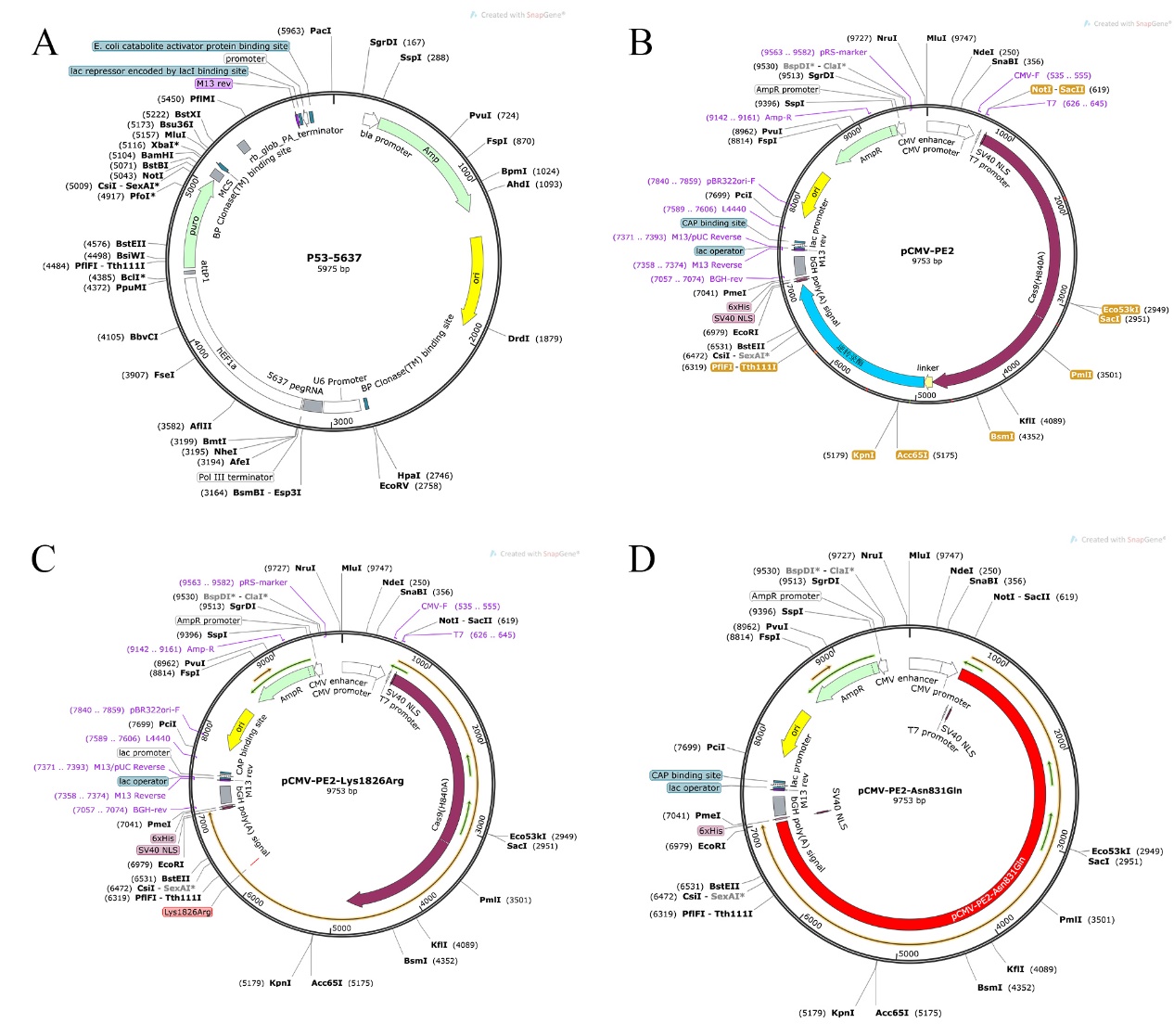
**

**Figure S2: Related plasmids.** (A) pZDonor_Seq1-U6-5637 pegRNA-terminator-hEF1a-Puro-Seq2. (B) pCMV-PE2. (C) pCMV-PE2-Lys1826Arg. (D) pCMV-PE2-Asn831Gln

**
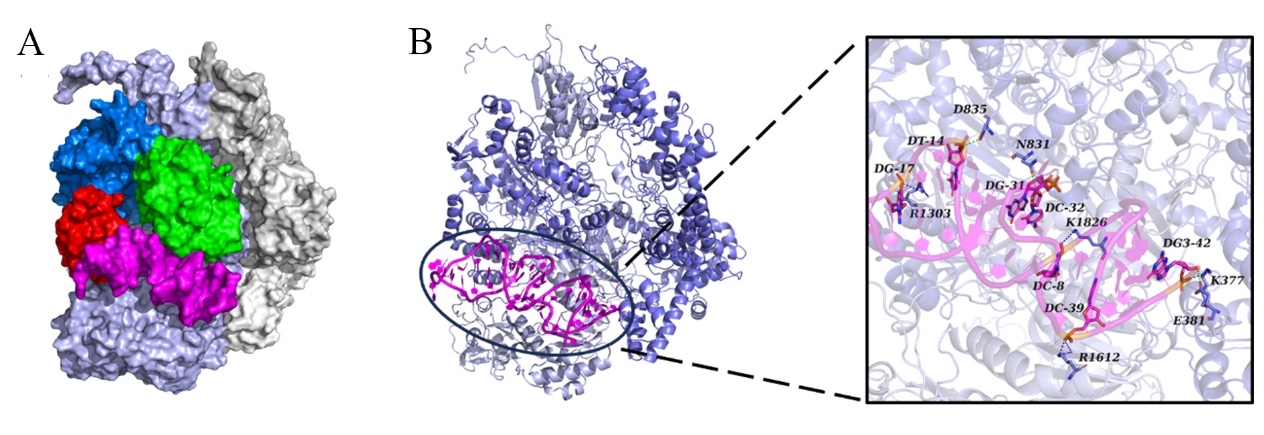
**

**Figure S3. Binding pose and interactions of PE2 with aptamer2.** (A)The surface representation of PE2 and aptamer2. (B) The ribbon diagram of PE2 and aptamer2. The PE2 fusion protein is colored in light and dark purple and aptamer 2 is colored in violet. The residues in PE2 and aptamer3 are depicted as purple and violet sticks, respectively. The hydrogen bond and salt bridge interactions are depicted as green and blue dashed lines, respectively.


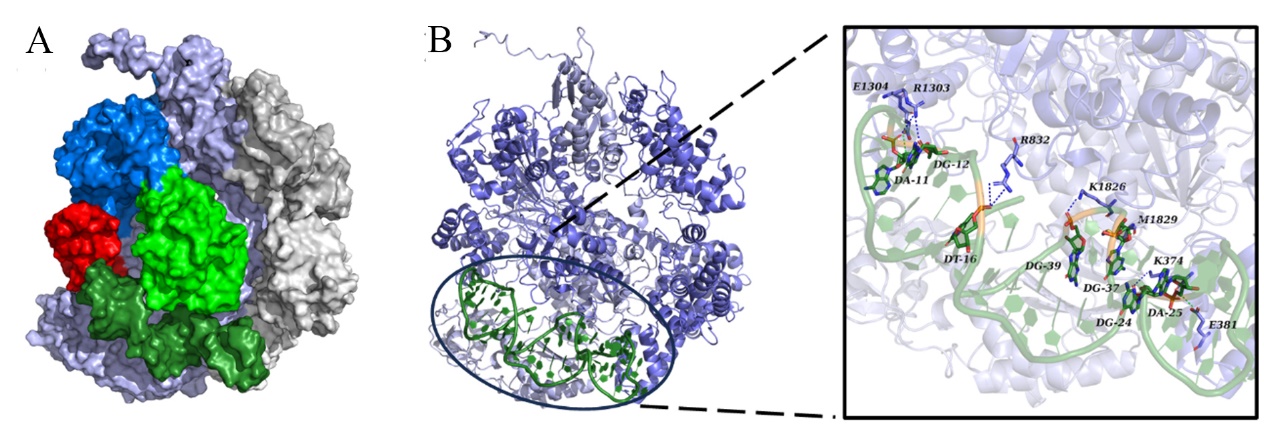


**Figure S4. Binding pose and interactions of PE2 with aptamer3.** (A)The surface representation of PE2 and aptamer3. (B) The ribbon diagram of PE2 and aptamer3. The PE2 fusion protein is colored in light and dark purple and aptamer3 is colored in green. The residues in PE2 and aptamer3 are depicted as purple and green sticks, respectively. The hydrogen bond and salt bridge interactions are depicted as green and blue dashed lines, respectively.


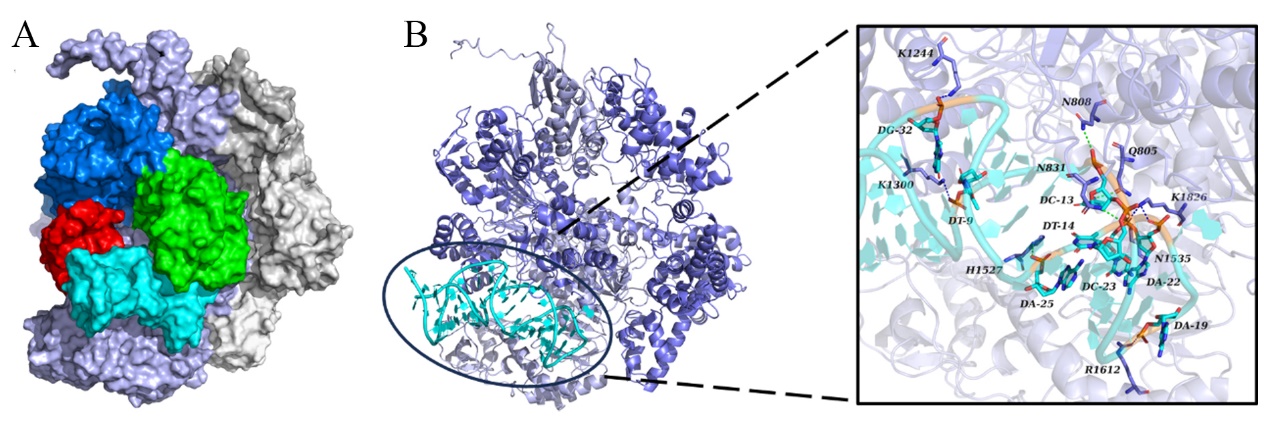


**Figure S5. Binding pose and interactions of PE2 with aptamer4.** (A)The surface representation of PE2 and aptamer4. (B) The ribbon diagram of PE2 and aptamer4. The PE2 fusion protein is colored in light and dark purple and aptamer4 is colored in tiffany blue. The residues in PE2 and aptamer4 are depicted as purple and tiffany blue sticks, respectively. The hydrogen bond and salt bridge interactions are depicted as green and blue dashed lines, respectively.


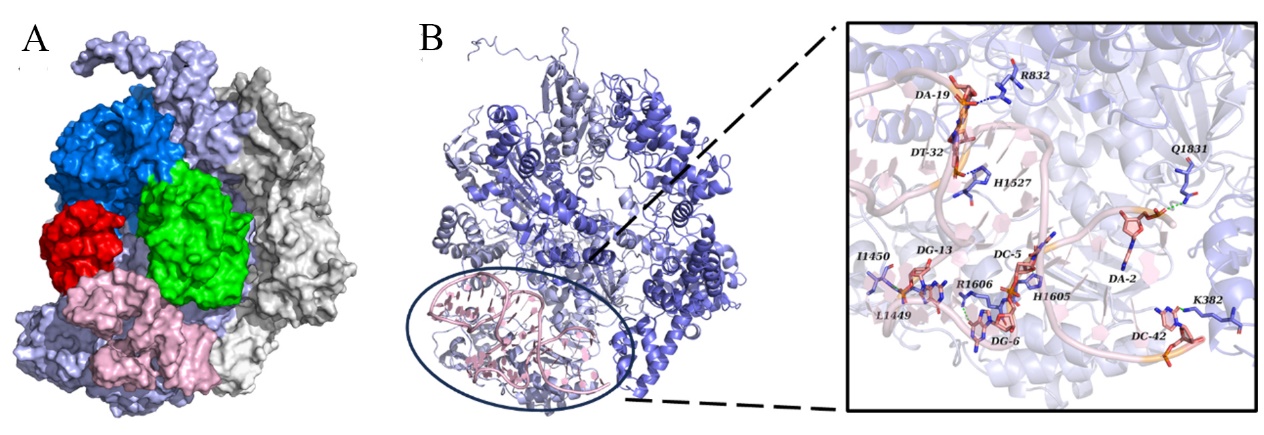


**Figure S6. Binding pose and interactions of PE2 with aptamer5.** (A)The surface representation of PE2 and aptamer5. (B) The ribbon diagram of PE2 and aptamer5. The PE2 fusion protein is colored in light and dark purple and aptamer5 is colored in pink. The residues in PE2 and aptamer5 are depicted as purple and pink sticks, respectively. The hydrogen bond and salt bridge interactions are depicted as green and blue dashed lines, respectively.


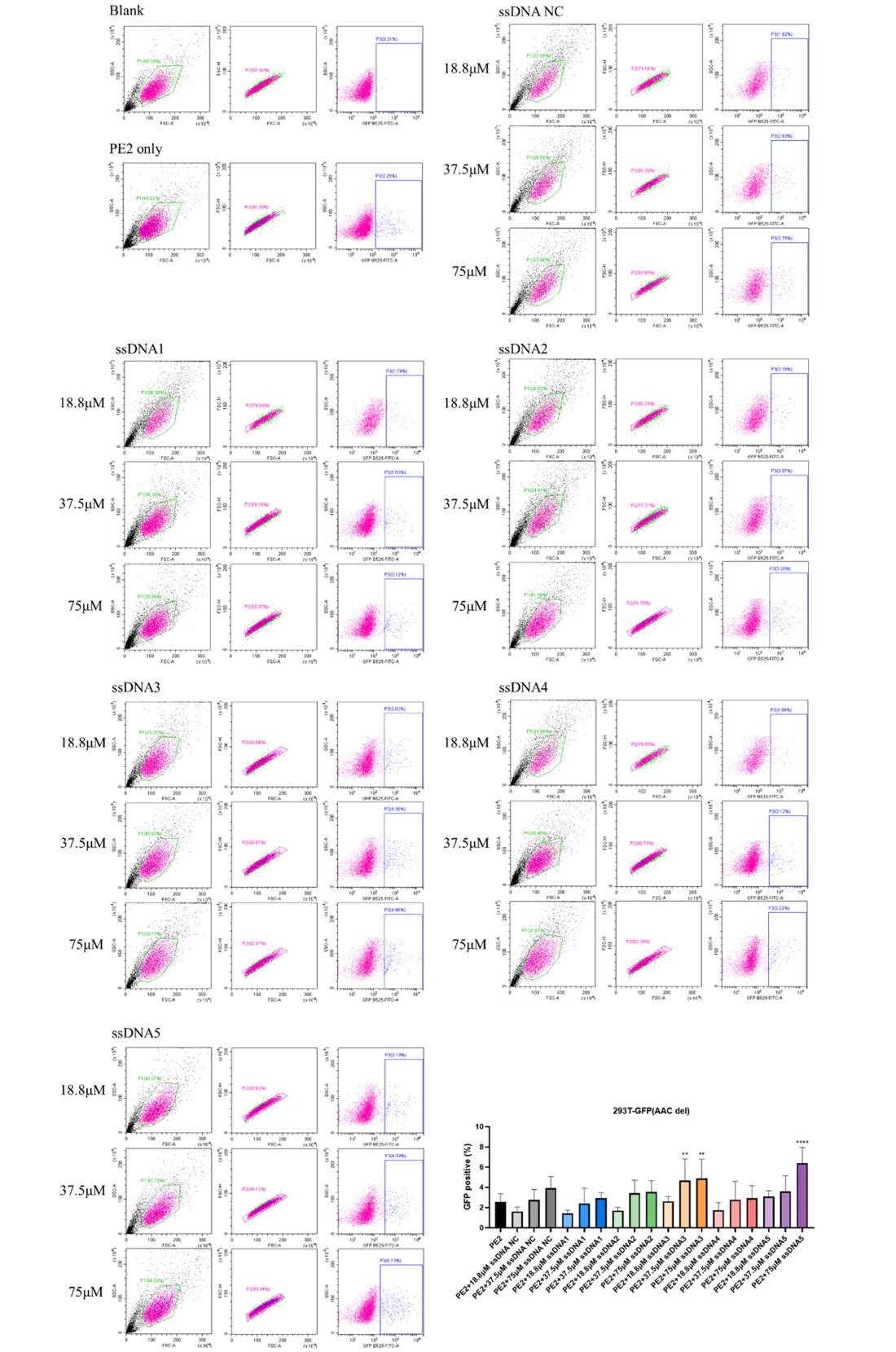


**Figure S7: Representative flow cytometry results of the GFP repairing efficiency under different aptamer treatments.** Aptamers with concentration gradient of 18.8/37.5/75μM were administered to the HEK293T (GFP del AAC) cells. The flow cytometry results represented PE2 only and PE2+aptamer groups on their efficiencies for GFP restoration via small insertion. Data are presented as means ± SD from at least three independent experiments (*P < 0.05, **P < 0.01, ****P < 0.0001).


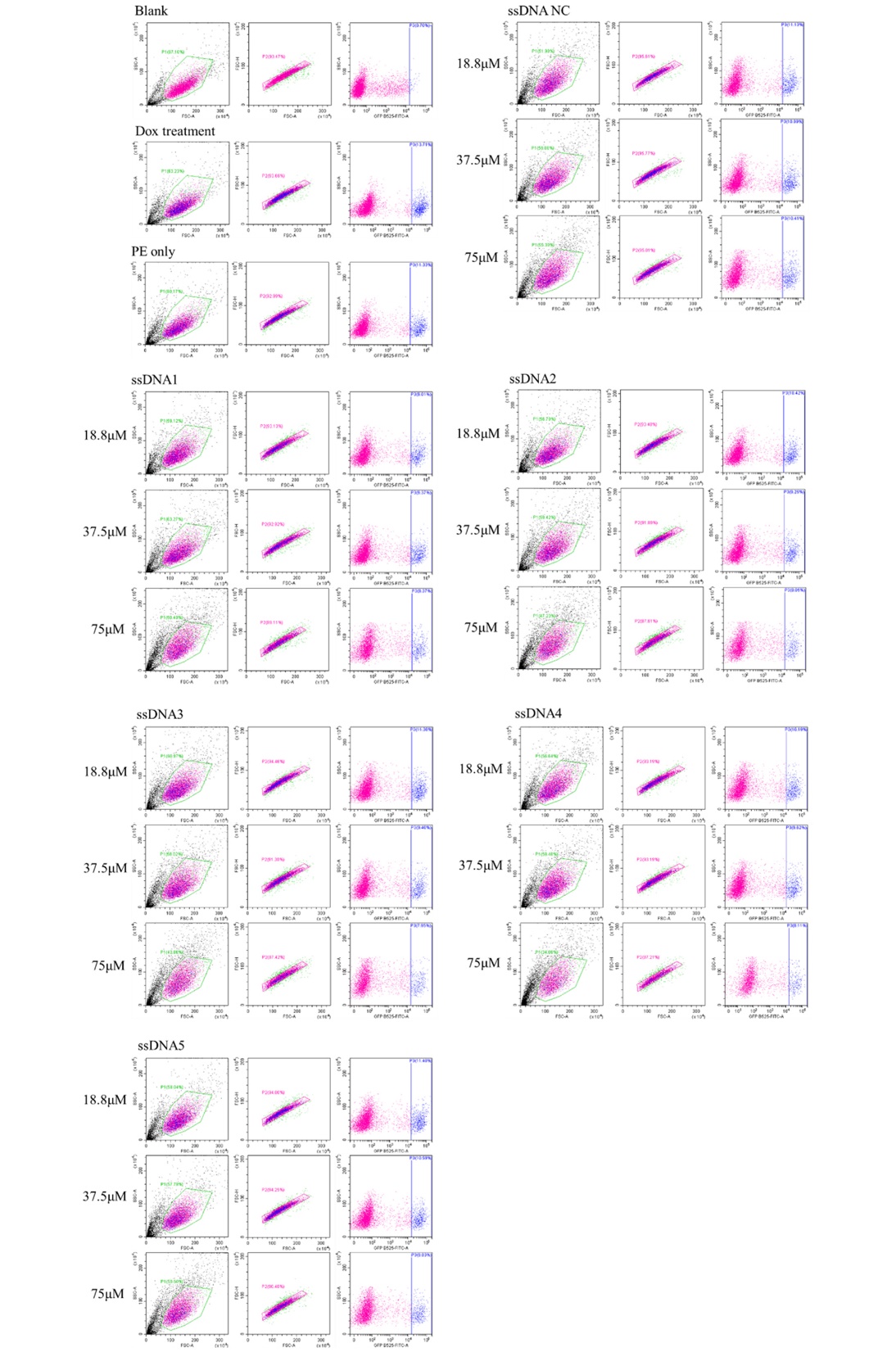


**Figure S8: Representative flow cytometry results of the EGFP destruction efficiency under different aptamer treatments.** Aptamers with concentration gradient of 18.8/37.5/75μM were administered to the HEK293T-411 cells. The flow cytometry results represented PE2 only and PE2+aptamer groups on their efficiencies for EGFP destruction via small insertion, which led to a frameshift and an early STOP codon. DOX treatment group were used as the positive control.


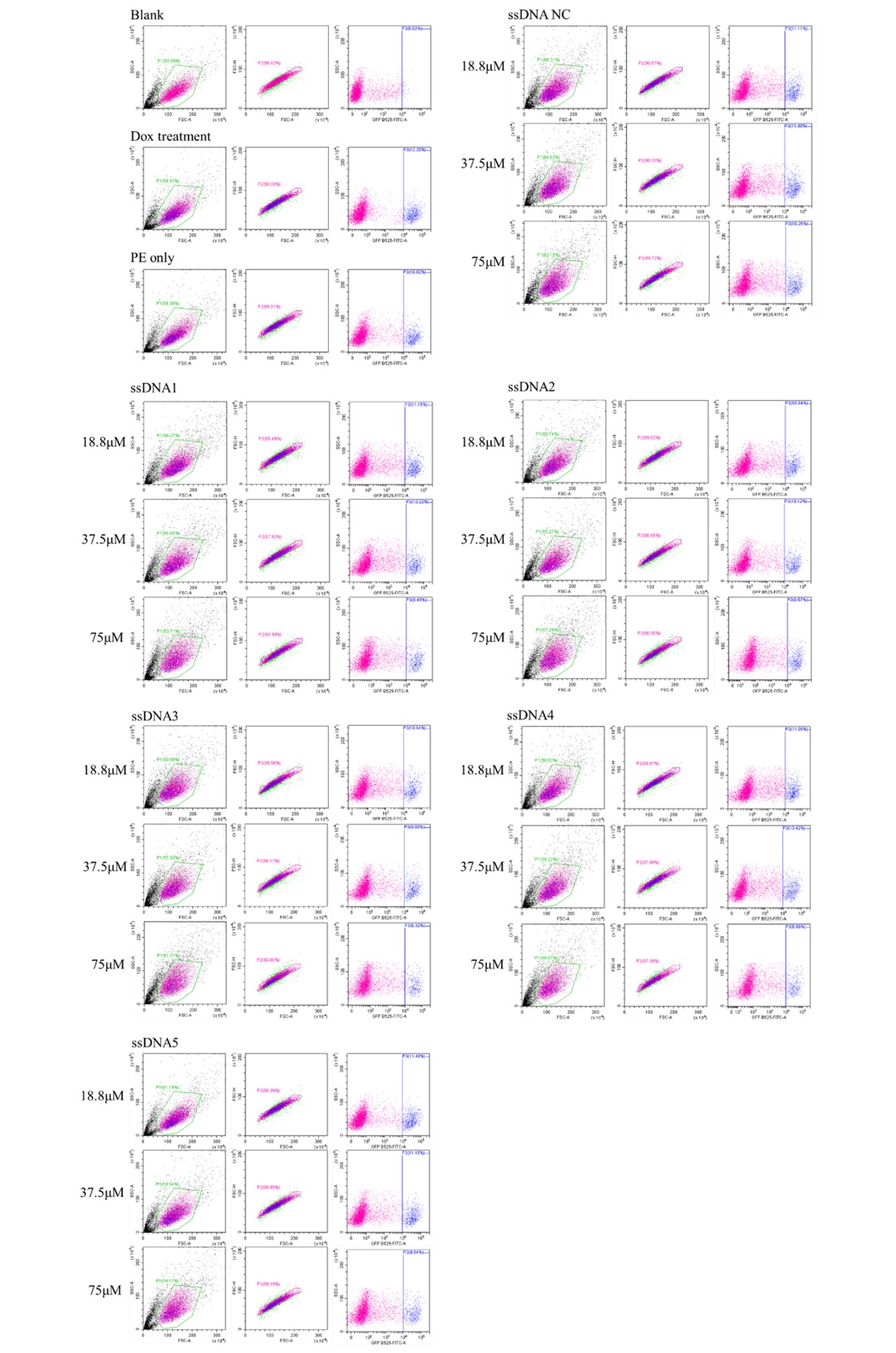


**Figure S9: Representative flow cytometry results of the EGFP destruction efficiency under different aptamer treatments.** Aptamers with concentration gradient of 18.8/37.5/75μM were administered to the HEK293T-411 cells. The flow cytometry results represented PE2 only and PE2+aptamer groups on their efficiencies for EGFP destruction via small deletion, which led to a frameshift and an early STOP codon. DOX treatment group were used as the positive control.


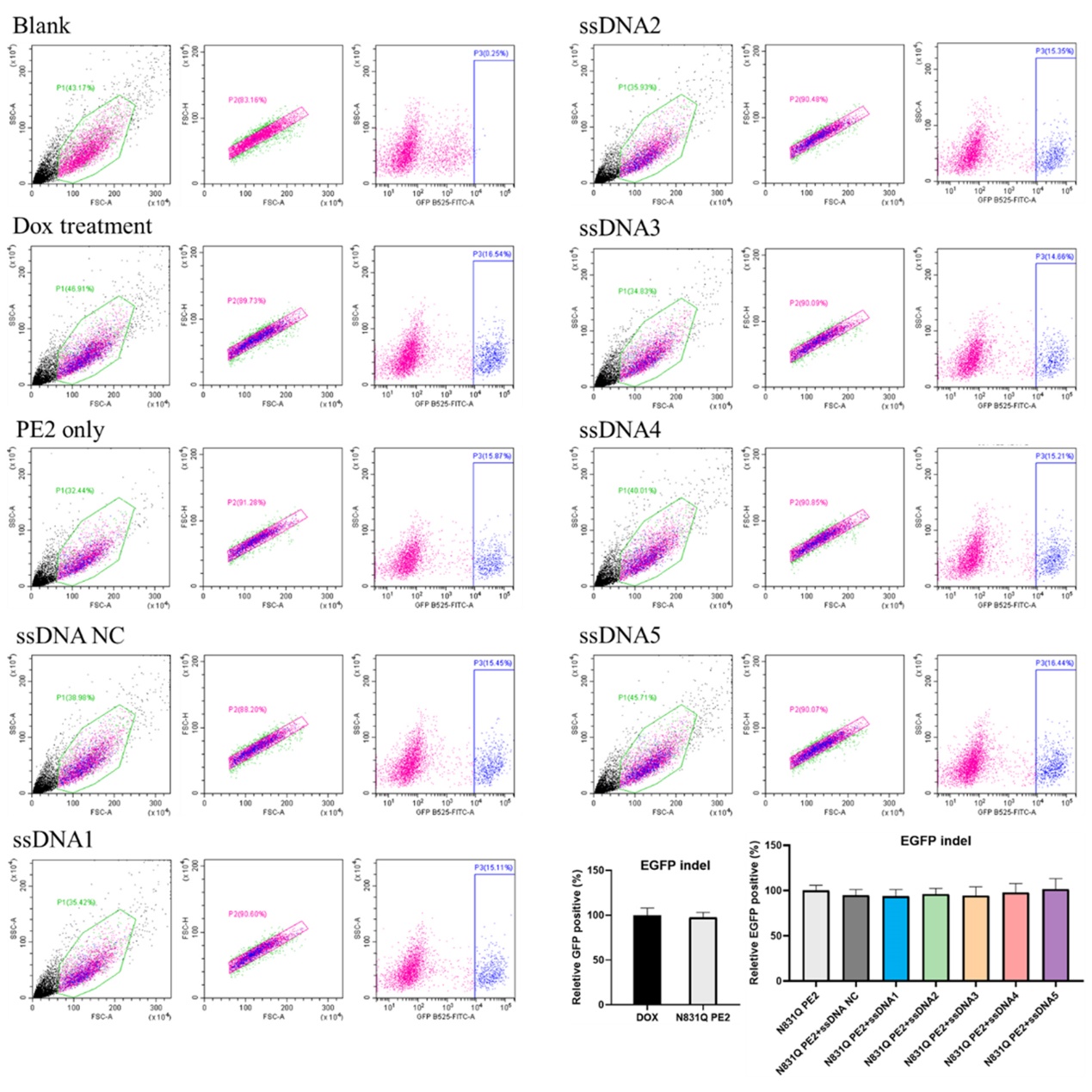


**Figure S10: Representative flow cytometry results on the effect of N831Q PE2 mutant on EGFP target editing.** The flow cytometry results compared the wild PE2, N831Q PE2 mutant, and N831Q PE2+aptamer groups on their efficiencies for EGFP destruction via small insertion, which led to a frameshift and an early STOP codon. DOX treatment group were presented as a positive control. Data are presented as means ± SD from at least three independent experiments.


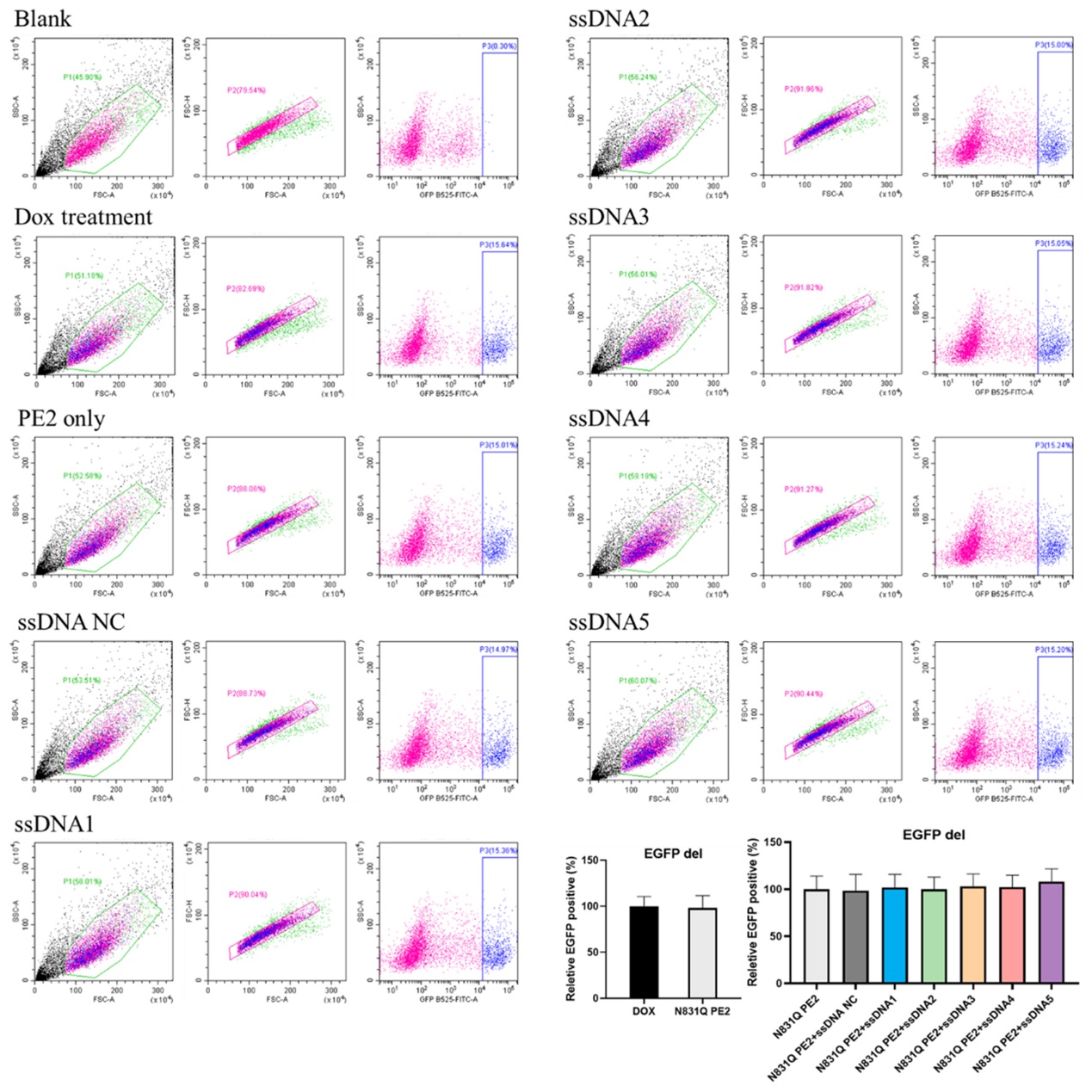


**Figure S11: Representative flow cytometry results on the effect of N831Q PE2 mutant on EGFP target editing.** The flow cytometry results compared the wild PE2, N831Q PE2 mutant, and N831Q PE2+aptamer groups on their efficiencies for EGFP destruction via small deletion, which led to a frameshift and an early STOP codon. DOX treatment group were presented as a positive control. Data are presented as means ± SD from at least three independent experiments.


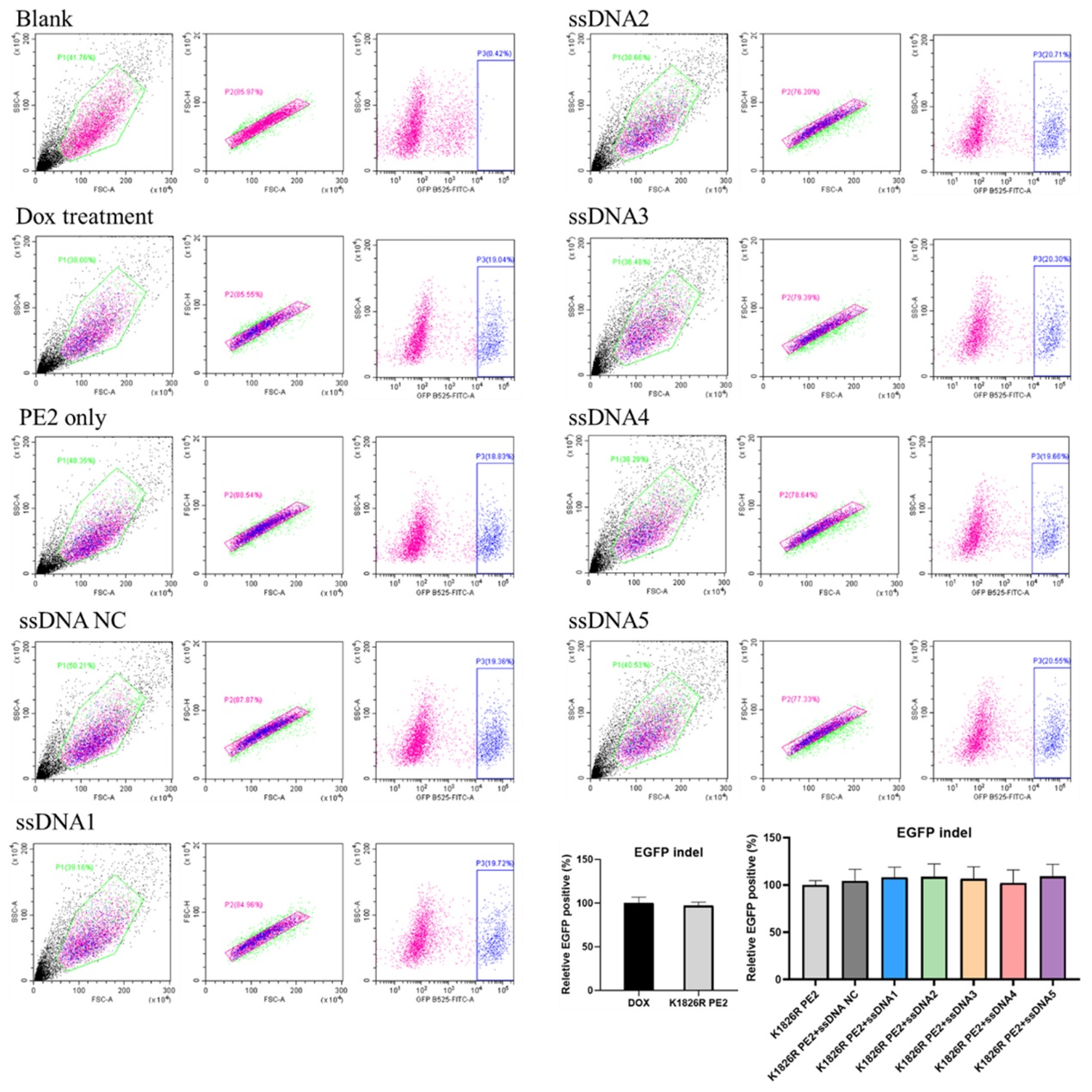


**Figure S12: Representative flow cytometry results on the effect of K1826R PE2 mutant on EGFP target editing.** The flow cytometry results compared the wild PE2, K1826R PE2 mutant, and K1826R PE2+aptamer groups on their efficiencies for EGFP destruction via small insertion, which led to a frameshift and an early STOP codon. DOX treatment group were presented as a positive control. Data are presented as means ± SD from at least three independent experiments.


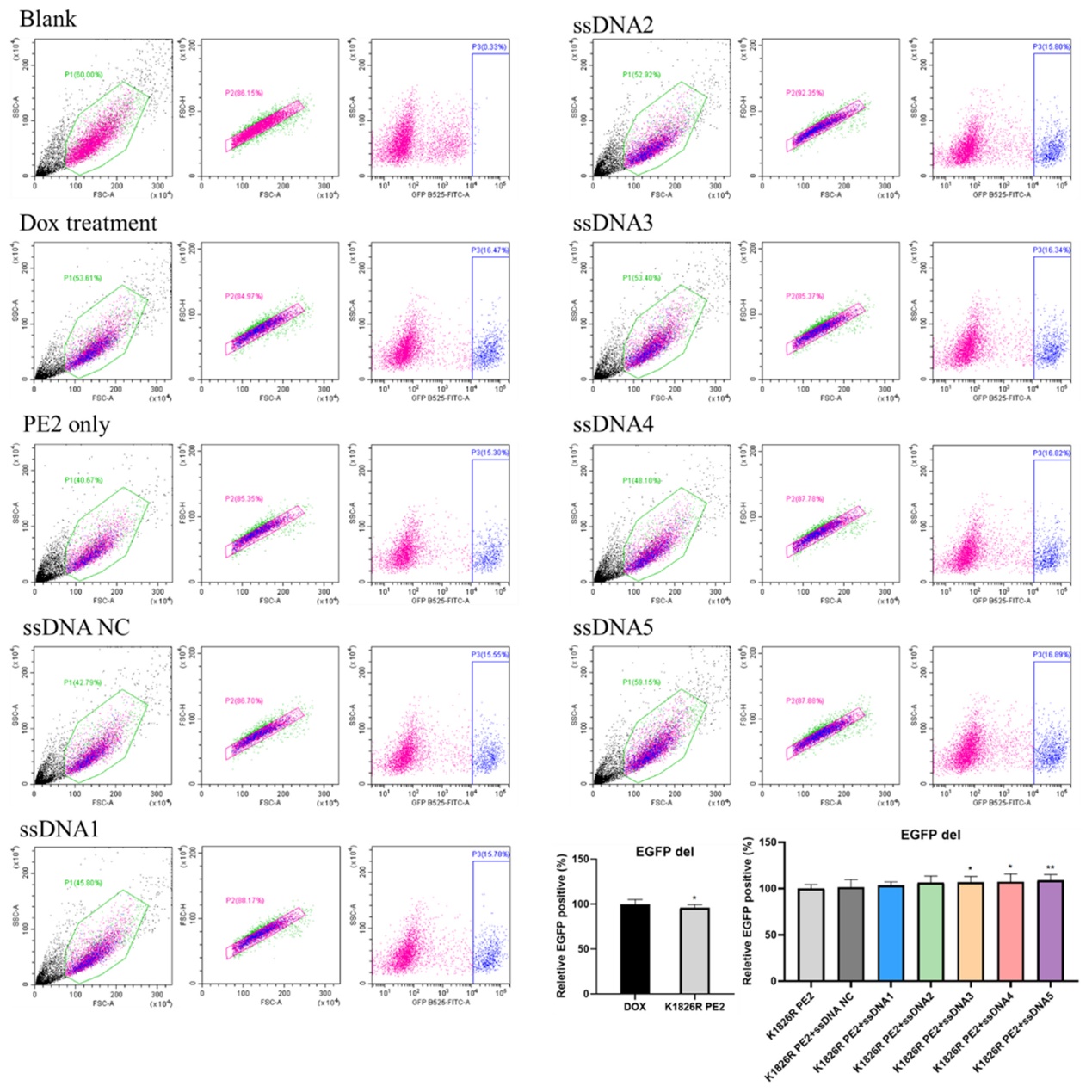


**Figure S13: Representative flow cytometry results on the effect of K1826R PE2 mutant on EGFP target editing.** The flow cytometry results compared the wild PE2, K1826R PE2 mutant, and K1826R PE2+aptamer groups on their efficiencies for EGFP destruction via small deletion, which led to a frameshift and an early STOP codon. DOX treatment group were presented as a positive control. Data are presented as means ± SD from at least three independent experiments (*P < 0.05, **P < 0.01).

**
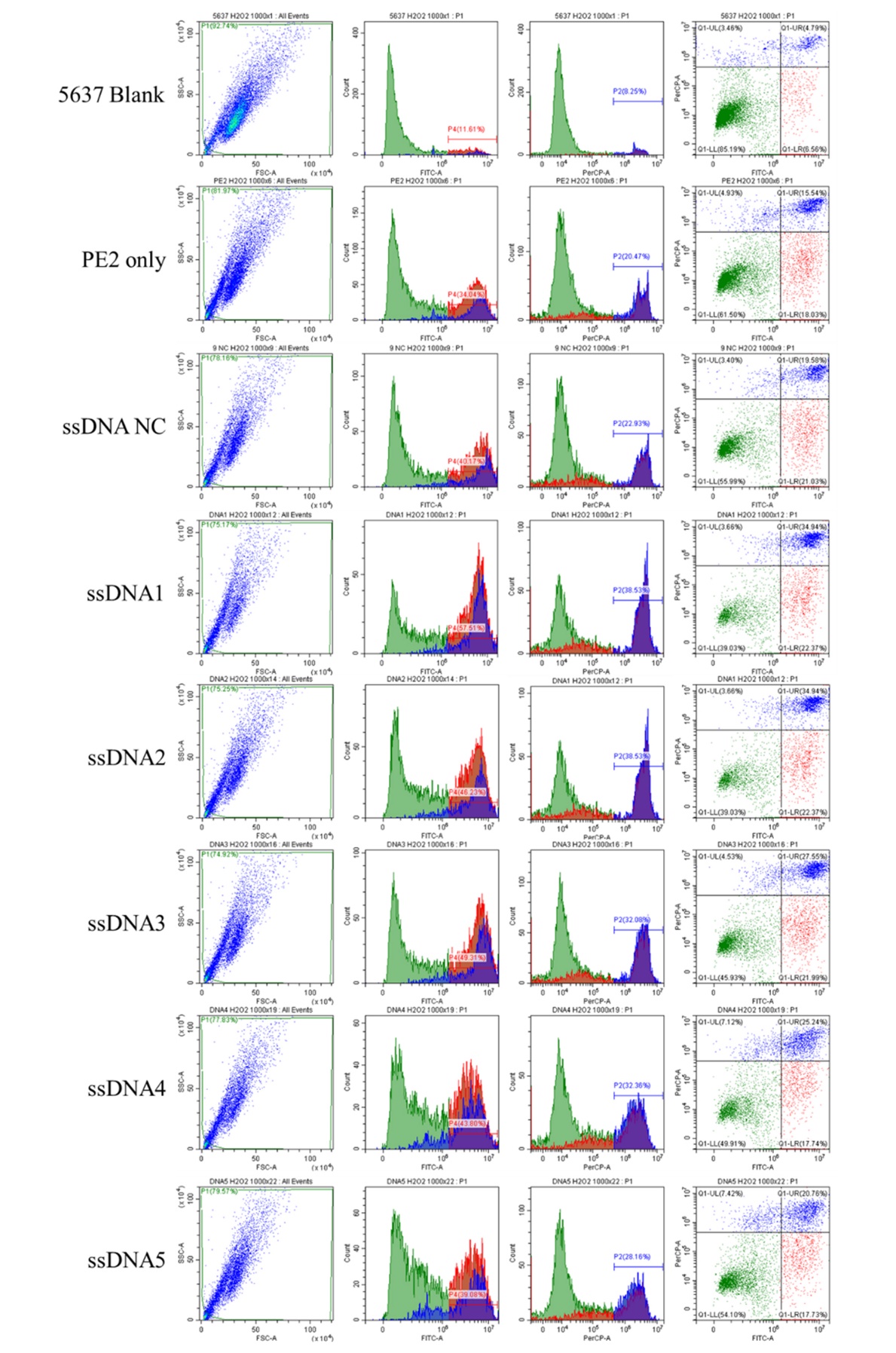
**

**Figure S14: Aptamer-PE2 system promoted the apoptosis in 5637 p53 deficient cells.** The aptamers promoted the PE2 editing and enhanced the wild-type p53 restoration, which led to an increased apoptosis under H_2_O_2_ stress stimulus. Quantified results of the apoptosis were assessed via flow cytometry. The blank group acted as the negative control for the PE2 group, and the ssDNA NC group acted as the negative control for the Cas9-specific aptamers. Then, aptamers were assessed for their ability to interfere with PE2 function.

**
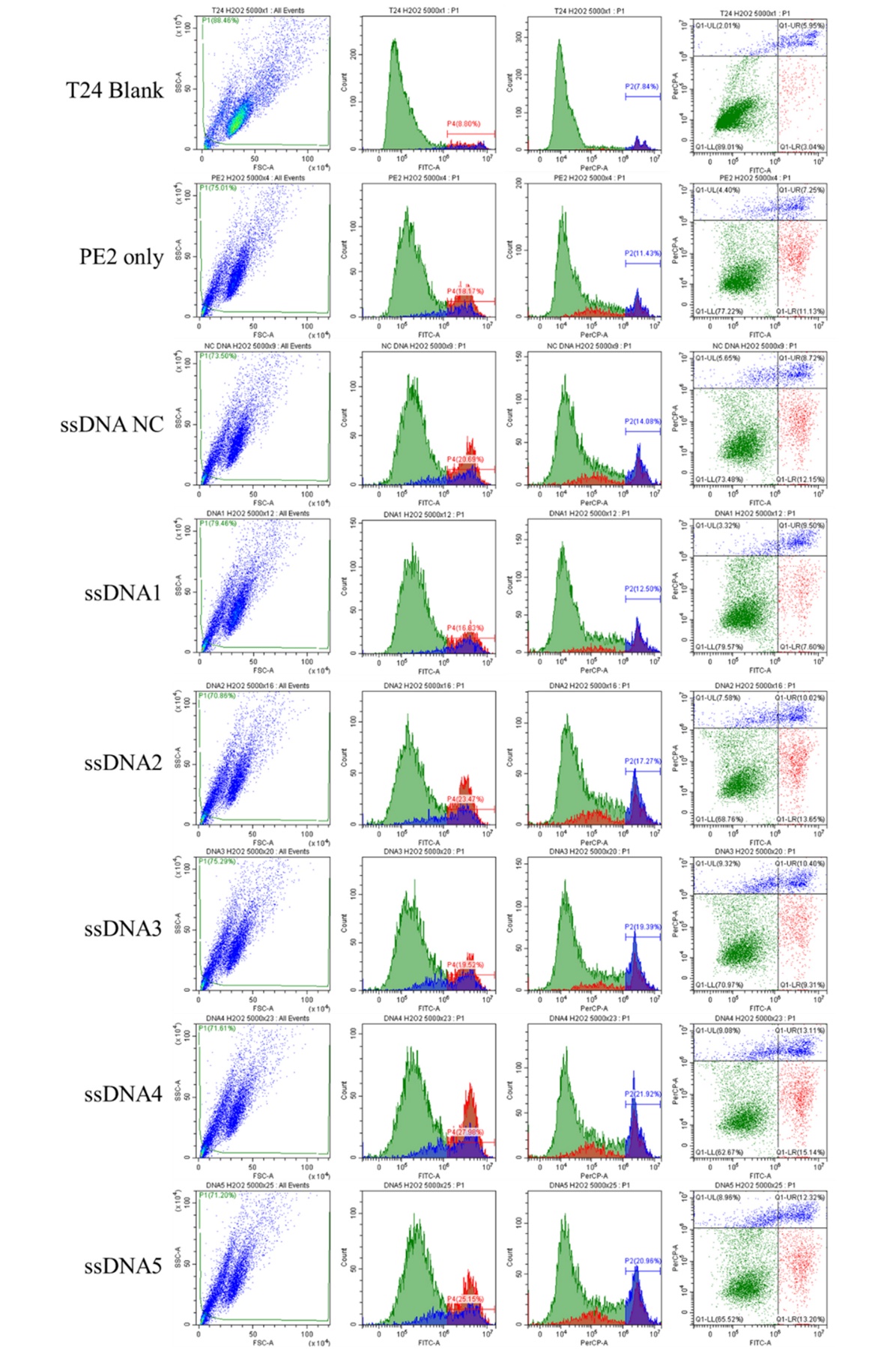
**

**Figure S15: Aptamer-PE2 system promoted the apoptosis in T24 p53 deficient cells.** The aptamers promoted the PE2 editing and enhanced the wild-type p53 restoration, which led to an increased apoptosis under H_2_O_2_ stress stimulus. Quantified results of the apoptosis were assessed via flow cytometry. The blank group acted as the negative control for the PE2 group, and the ssDNA NC group acted as the negative control for the Cas9-specific aptamers. Then, aptamers were assessed for their ability to interfere with PE2 function.


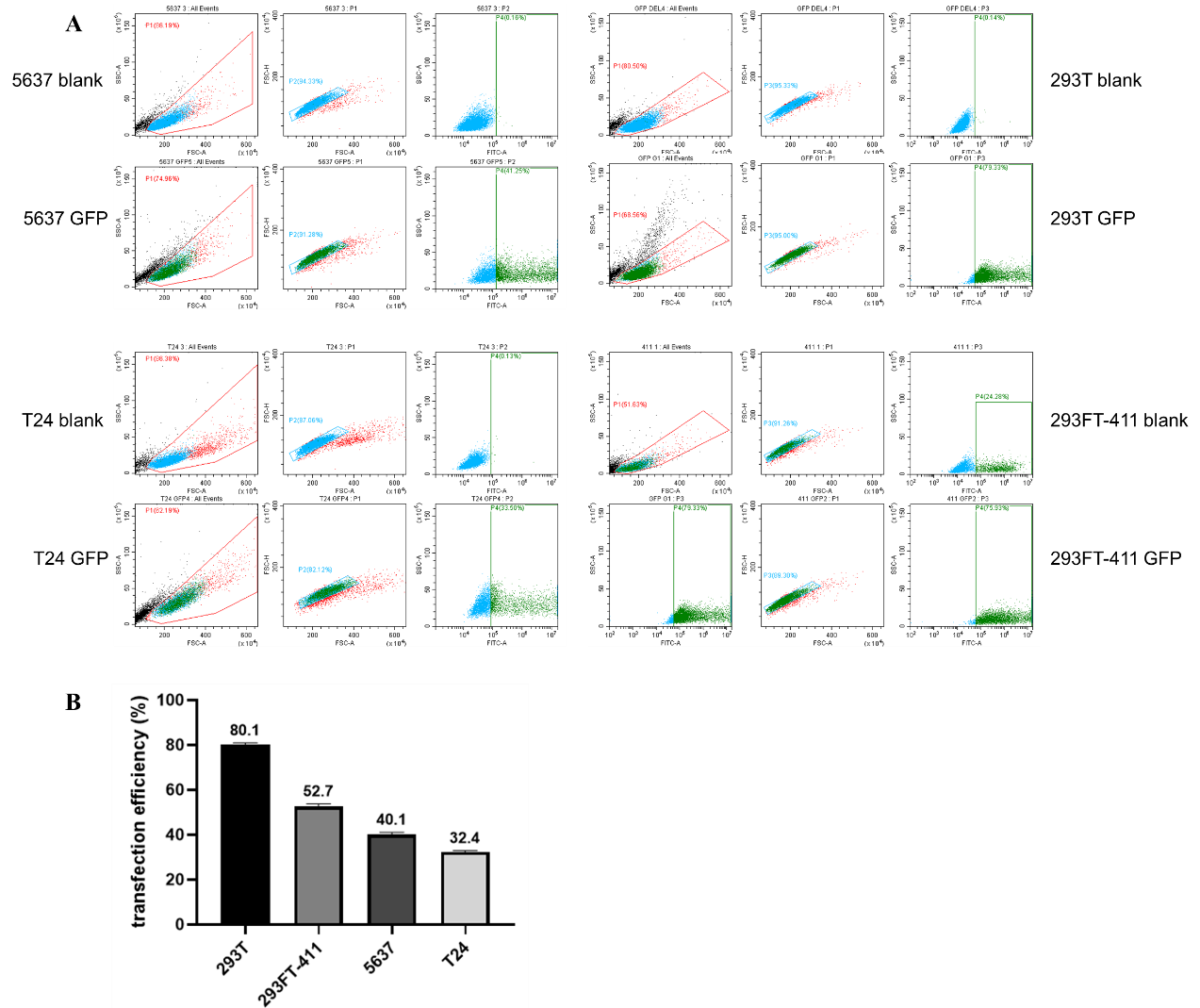


**Figure S16: Transfection Efficiency of Lipofectamine 3000 in Different Cell Lines.** (A) GFP reporter plasmid was transfected into each cell line at a concentration of 1 μg/μL, and the transient transfection efficiency across different cell lines was determined by flow cytometry. (B) Editing efficiency was normalized using non-transfected blank cells as the negative control. Data are presented as mean ± SD from at least three independent experiments.

**Table S1: ssDNA related sequences.** Astral mark (*) stands for phosphorothioates modification.

| **Name** | **5’ to 3’** |
| --- | --- |
| **NC aptamer** | G*G*G*AACACGACGGCGCTGTGGAAGGCACAGTACAGGCGTGTG*G*G*C |
| **aptamer 1** | A*A*C*ACGACGTGTCTGTGAATCACACAGTCCAACAATGTC*G*T*G |
| **aptamer 2** | A*A*C*ACGACTCCACTGTGCGTCGCGAACTCTGCCACGGTC*G*T*G |
| **aptamer 3** | A*A*C*ACGACGCAGCTGTGGCCAGCGAACTGTGTGGGAGTG*G*G*C |
| **aptamer 4** | A*A*G*CCCACTGGTCTGTGGACAACGAACGCCGGTGTGGTC*G*T*G |
| **aptamer 5** | A*A*C*ACGACACAAGTTCGAAGGACGAACGTCATGCAAGTG*G*G*C |

**Table S2.** **The contact list between PE2 with aptamer 1.**

| **Chain 1** | **Residue** | **Chain 2** | **Residue** | **Interaction type** |
| --- | --- | --- | --- | --- |
| PE2 | Glu387. OE2 | aptamer1 | DA1. O5' | Hydrogen bond |
| PE2 | Lys797. O | aptamer1 | DG9. OP1 | Hydrogen bond |
| PE2 | Arg1870. NE | aptamer1 | DT10. OP2 | Hydrogen bond |
| PE2 | Asp1525. O | aptamer1 | DT20. N3 | Hydrogen bond |
| PE2 | Asn808. ND2 | aptamer1 | DC23. O5' | Hydrogen bond |
| PE2 | Lys377. NZ | aptamer1 | DG37. OP1 | Salt bridge |
| PE2 | Arg220. NH1 | aptamer1 | DC39. OP1 | Hydrogen bond |

**Table S3. The contact list between PE2 and aptamer2**

| **Chain 1** | **Residue** | **Chain 2** | **Residue** | **Interaction type** |
| --- | --- | --- | --- | --- |
| PE2 | Lys1826. NZ | aptamer2 | DC8. OP2 | Salt bridge |
| PE2 | Asp835. OD2 | aptamer2 | DT14. C5' | Hydrogen bond |
| PE2 | Arg1303. NH2 | aptamer2 | DG17. OP1 | Salt bridge |
| PE2 | Asn831. OD1 | aptamer2 | DG31. C2' | Hydrogen bond |
| PE2 | Asn831. OD1 | aptamer2 | DC32. C5' | Hydrogen bond |
| PE2 | Arg1612.NE, NH1 | aptamer2 | DC39. OP1 | Salt bridge |
| PE2 | Lys377. NZ | aptamer2 | DG42. OP1 | Salt bridge |
| PE2 | Glu381. OE1 | aptamer2 | DG42. O3' | Hydrogen bond |

**Table S4. The contact list between PE2 and aptamer3**

| **Chain 1** | **Residue** | **Chain 2** | **Residue** | **Interaction type** |
| --- | --- | --- | --- | --- |
| PE2 | Glu1304.OE1 | aptamer3 | DA11.C3' | Hydrogen bond |
| PE2 | Glu381.OE1 | aptamer3 | DA25.C5' | Hydrogen bond |
| PE2 | Met1829.O | aptamer3 | DG37.C1' | Hydrogen bond |
| PE2 | Arg1303.NH2 | aptamer3 | DA11.OP1 | Salt bridge |
| PE2 | Arg1303.NH2 | aptamer3 | DG12.OP1 | Salt bridge |
| PE2 | Arg832.NH1, NH2 | aptamer3 | DT16.OP1 | Salt bridge |
| PE2 | Lys374.NZ | aptamer3 | DG24.OP1 | Salt bridge |
| PE2 | Lys1826.NZ | aptamer3 | DG39.OP1 | Salt bridge |

**Table S5. The contact list between PE2 and aptamer4**

| **Chain 1** | **Residue** | **Chain 2** | **Residue** | **Interaction type** |
| --- | --- | --- | --- | --- |
| PE2 | Gln805.OE1 | aptamer4 | DC13.N4 | Hydrogen bond |
| PE2 | Asn808.ND2 | aptamer4 | DC13.OP2 | Hydrogen bond |
| PE2 | Asn831.ND2 | aptamer4 | DT14.O5' | Hydrogen bond |
| PE2 | Asn1535.ND2 | aptamer4 | DA22.O3' | Hydrogen bond |
| PE2 | His1527.ND1 | aptamer4 | DA25.OP2 | Hydrogen bond |
| PE2 | Lys1300.NZ | aptamer4 | DT9.OP2 | Salt bridge |
| PE2 | Arg1612.NH1 | aptamer4 | DA19.OP2 | Salt bridge |
| PE2 | Lys1826.NZ | aptamer4 | DA22.OP1 | Salt bridge |
| PE2 | Lys1826.NZ | aptamer4 | DC23.OP1 | Salt bridge |
| PE2 | Lys1244.NZ | aptamer4 | DG32.OP2 | Salt bridge |

**Table S6. The contact list between PE2 and aptamer5**

| **Chain 1** | **Residue** | **Chain 2** | **Residue** | **Interaction type** |
| --- | --- | --- | --- | --- |
| PE2 | Gln1831.NE2 | aptamer5 | DA2.OP1 | Hydrogen bond |
| PE2 | His1605.ND1 | aptamer5 | DC5.OP2 | Hydrogen bond |
| PE2 | Arg1606.NH1 | aptamer5 | DG6.O6 | Hydrogen bond |
| PE2 | Leu1449.CA | aptamer5 | DG13.OP2 | Hydrogen bond |
| PE2 | Ile1450.N | aptamer5 | DG13.OP2 | Hydrogen bond |
| PE2 | Lys382.NZ | aptamer5 | DC42.O2 | Hydrogen bond |
| PE2 | His1527.NE2 | aptamer5 | DT32.OP2 | Salt bridge |
| PE2 | Arg832.NH1 | aptamer5 | DA19.OP2 | Salt bridge |
